# (*S*,*Z*)-3-Phenyl-2-[(1,1,1-tri­chloro-7-meth­oxy-2,7-dioxohept-3-en-4-yl)amino]­propanoic acid monohydrate

**DOI:** 10.1107/S1600536814000154

**Published:** 2014-01-18

**Authors:** Alex Fabiani Claro Flores, Juliano Rosa de Menezes Vicenti, Lucas Pizzuti, Patrick Teixeira Campos

**Affiliations:** a Escola de Quimica e Alimentos, Universidade Federal do Rio Grande, Av. Italia, km 08, Campus Carreiros, 96203-900 Rio Grande, RS, Brazil; b Universidade Federal da Grande Dourados, UFGD, CEP 79825-070 Dourados, MS, Brazil; c Instituto Federal Farroupilha, Campus Júlio de Castilhos, CEP 98130-000, Júlio de Castilhos, RS, Brazil

## Abstract

In the title compound, C_17_H_18_Cl_3_NO_5_·H_2_O, intra­molecular N—H⋯O and C—H⋯Cl hydrogen bonds form *S*(6) and *S*(5) ring motifs, respectively. The chiral organic mol­ecule is connected to the solvent water mol­ecule by a short O—H⋯O hydrogen bond. In the crystal, a weak C—H⋯Cl inter­action connects the organic mol­ecules along [100] while the water mol­ecules act as bridges between the organic mol­ecules in both the [100] and [010] directions, generating layers parallel to the *ab* plane.

## Related literature   

For the synthesis of the title compound and a similar crystal structure, see: Flores *et al.* (2008 ▶). For information about levulinic acid and the biological properties of its derivatives, see: Flores *et al.* (2013 ▶); Hachuła *et al.* (2013 ▶); Lo & Ng (2008 ▶). For short inter­molecular hydrogen-bond inter­actions, see: Pojarová *et al.* (2010 ▶). For intra­molecular hydrogen-bonding systems, see: da Costa *et al.* (2013 ▶).
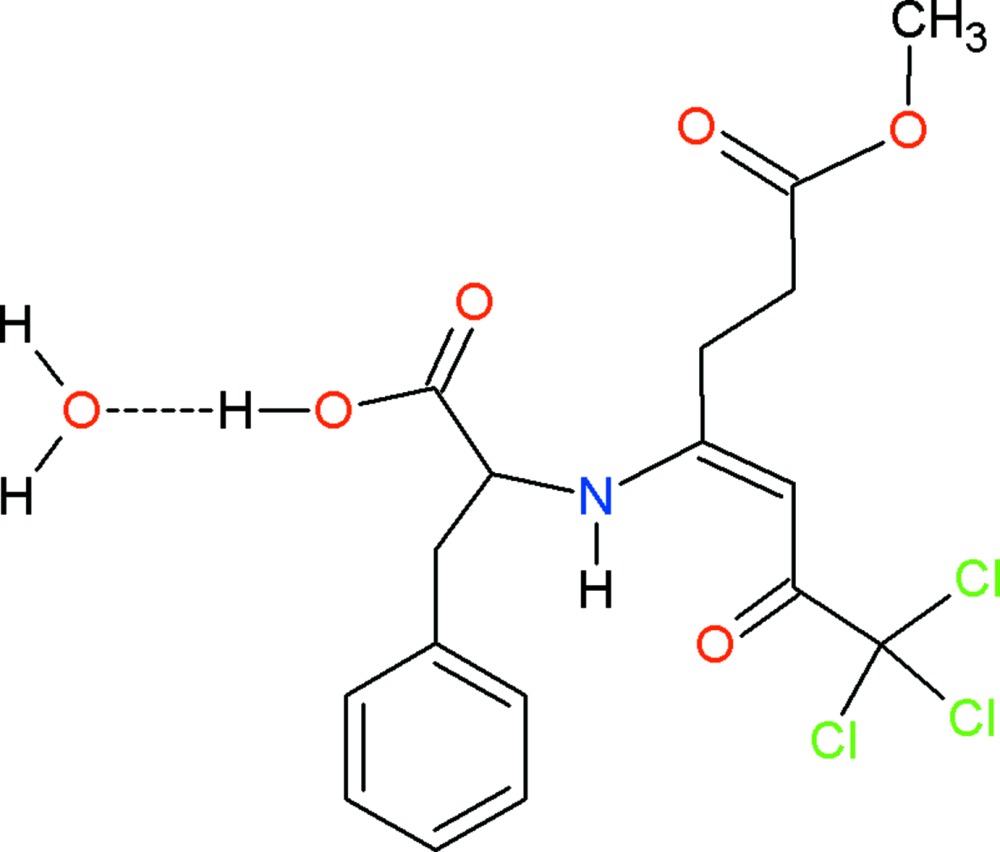


## Experimental   

### 

#### Crystal data  

C_17_H_18_Cl_3_NO_5_·H_2_O
*M* *_r_* = 440.69
Triclinic, 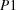

*a* = 5.6684 (16) Å
*b* = 8.601 (3) Å
*c* = 10.336 (3) Å
α = 87.720 (19)°
β = 85.696 (17)°
γ = 85.649 (17)°
*V* = 500.8 (2) Å^3^
*Z* = 1
Mo *K*α radiation
μ = 0.49 mm^−1^
*T* = 296 K
0.98 × 0.30 × 0.12 mm

#### Data collection  

Bruker APEXII CCD diffractometer
Absorption correction: Gaussian (*XPREP*; Bruker, 2006 ▶) *T*_min_ = 0.881, *T*_max_ = 1
13424 measured reflections
6020 independent reflections
4784 reflections with *I* > 2σ(*I*)
*R*_int_ = 0.024

#### Refinement  

*R*[*F* ^2^ > 2σ(*F* ^2^)] = 0.040
*wR*(*F* ^2^) = 0.105
*S* = 1.04
6020 reflections
256 parameters
3 restraints
H atoms treated by a mixture of independent and constrained refinement
Δρ_max_ = 0.41 e Å^−3^
Δρ_min_ = −0.32 e Å^−3^
Absolute structure: Flack parameter determined using 1984 quotients [(*I* ^+^)−(*I* ^−^)]/[(*I* ^+^)+(*I* ^−^)] (Parsons *et al.*, 2013 ▶)
Absolute structure parameter: 0.04 (2)

### 

Data collection: *APEX2* (Bruker, 2009 ▶); cell refinement: *SAINT* (Bruker, 2009 ▶); data reduction: *SAINT*; program(s) used to solve structure: *SHELXS97* (Sheldrick, 2008 ▶); program(s) used to refine structure: *SHELXL2013* (Sheldrick, 2008 ▶); molecular graphics: *DIAMOND* (Brandenburg, 2006 ▶); software used to prepare material for publication: *publCIF* (Westrip, 2010 ▶).

## Supplementary Material

Crystal structure: contains datablock(s) I. DOI: 10.1107/S1600536814000154/pk2509sup1.cif

Structure factors: contains datablock(s) I. DOI: 10.1107/S1600536814000154/pk2509Isup2.hkl

Supporting information file. DOI: 10.1107/S1600536814000154/pk2509Isup3.cml

CCDC reference:

Additional supporting information:  crystallographic information; 3D view; checkCIF report

## Figures and Tables

**Table 1:** Hydrogen-bond geometry (Å, °)

*D*—H⋯*A*	*D*—H	H⋯*A*	*D*⋯*A*	*D*—H⋯*A*
C6—H6*A*⋯Cl1^i^	0.97	2.94	3.774 (3)	145
O33—H33*B*⋯O91^ii^	0.87 (6)	1.89 (6)	2.766 (4)	177 (5)
N41—H41⋯O21	0.83 (5)	2.05 (6)	2.672 (3)	131 (5)
O33—H33*A*⋯O21^iii^	0.76 (6)	2.06 (6)	2.815 (3)	171 (6)
O92—H92⋯O33	0.89 (5)	1.66 (5)	2.542 (4)	175 (5)
C3—H3⋯Cl1	0.93	2.55	3.031 (3)	112

Symmetry codes: (i) 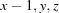; (ii) 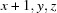; (iii) 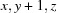.

**Fig. 1.**
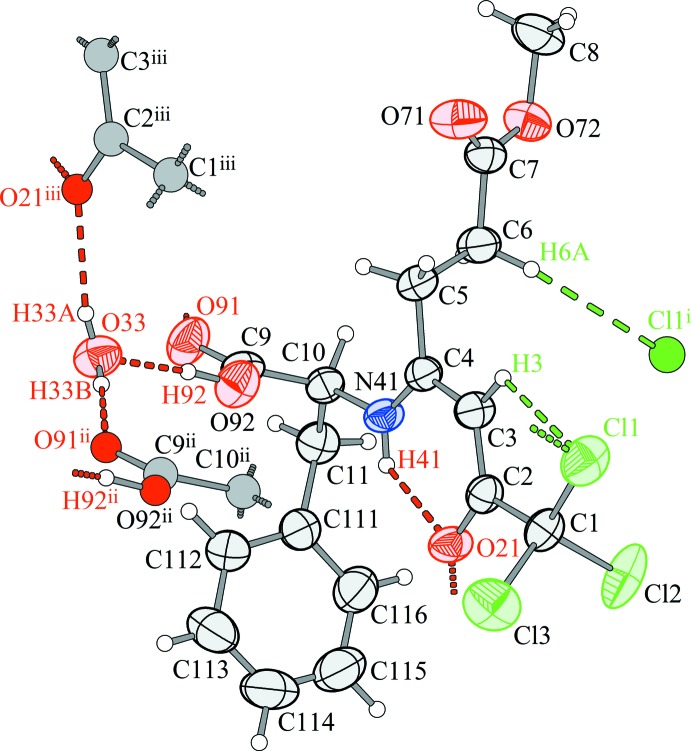
An ellipsoid plot (50% probability) showing the asymmetric unit. Hydrogen bonds are represented as dashed lines. Symmetry codes: (i) x–1, y, z; (ii) x + 1, y, z; (iii) x, y + 1, z.

**Fig. 2.**
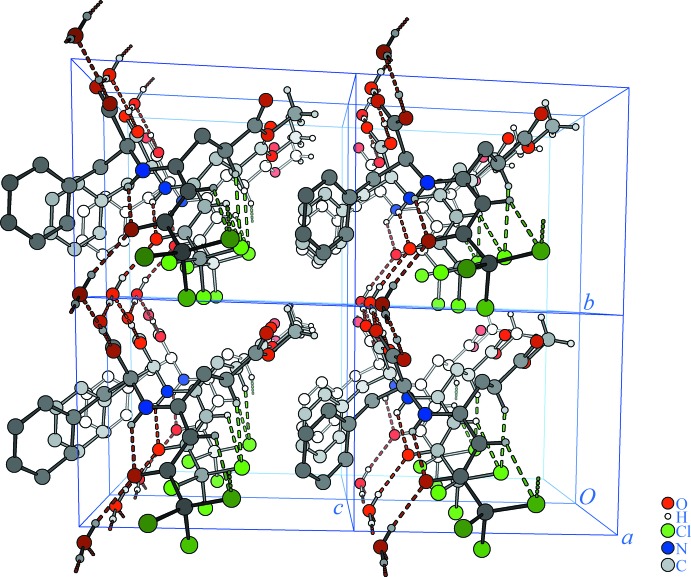
Packing of molecules along the [100] direction through intermolecular hydrogen bonds, represented with dashed lines. Some hydrogen atoms were omitted for clarity.

## supplementary crystallographic information

### 1. Comment

Dielectrophiles derived from levulinic acid (Hachuła *et al.*, 2013; Lo & Ng, 2008) belong to an important class of organic synthetic intermediates for the synthesis of a variety of heterocyclic compounds. Such precursors are used to produce pyrrolidinones, pyrrolones, pyrazoles and pyrimidines with very interesting biological activities (Flores *et al.*, 2008; Flores *et al.*, 2013). As a part of our studies, we report in this paper the crystal structure of (*S*,*Z*)-3-phenyl-2-(1,1,1-trichloro-7–2,7-dioxo-3-hepten- 4-ylamine)propanoic acid, obtained from the reaction between methyl 7,7,7-trichloro-4-methoxy-6-oxo-3-heptenoate and *L*-phenylalanine.

In the crystal structure of the title compound, the asymmetric unit is composed of the whole chiral organic molecule, C_17_H_18_Cl_3_NO_5_, connected to a water molecule (Fig.1). This connection consists of a short intermolecular hydrogen bond interaction involving the hydrogen atom of the carboxylic acid fragment [O92—H92···O33, 2.542 (4) Å; Pojarová *et al.*, 2010]. Additionally, *S*(6) and *S*(5) ring motifs are formed by two distinct intramolecular hydrogen bonding systems, N41—H41···O21 [2.672 (3) Å] and C3—H3···Cl1 [3.031 (3) Å], respectively, thereby stabilizing the structure (da Costa *et al.*, 2013).

There is also a weak C6—H6A···Cl1^i^ intermolecular interaction [3.774 (3) Å] connecting organic molecules along the [100] crystallographic direction. The water molecules act as a bridging element in the crystal structure by expanding its dimensionality in both [100] and [010] crystallographic directions. The intermolecular hydrogen bond interactions generate bidimensional layers parallel to the *ab* plane. Each atom of the water molecule is connected to different groups on adjacent organic molecules: carboxylic acid [O92—H92···O33, 2.542 (4) Å and O33—H33B···O91^ii^, 2.766 (4) Å] and ketone [O33—H33A···O21^iii^, 2.815 (3) Å]. Symmetry codes: (i) *x*–1, *y*, *z*; (ii) *x* + 1, *y*, *z*; (iii) *x*, *y* + 1, *z*. A super cell central projection of the crystal structure can be viewed in Fig. 2, which depicts a crystal packing diagram as viewed along the crystallographic *a* axis.

### 2. Experimental

To a stirred solution of methyl 7,7,7-trichloro-4-methoxy-6-oxo-3-heptenoate (5 mmol, 1.52 g) and *L*-phenylalanine (5.5 mmol, 0.91 g), at 25 °C, was added a solution of 1 mol·*L*^-1^ NaOH. There was an immediate formation of a yellow precipitate and the mixture was further stirred for 30 minutes. A solution of 50% HCl was added until the pH ≈ 1, when there was complete precipitation of the yellow solid. The solid was extracted with ethyl acetate, and this solution was dried over anhydrous MgSO_4_. The ethyl acetate was removed on a rotary evaporator to give the product as a yellow solid. Yield: 79%. m. p. 120 – 123 °C. ^1^H NMR (400 MHz, DMSO-D_6_, TMS): δ 2.17 (m, 2H, CH_2_), 2.44 (m, 2H, CH_2_), 3.06 (dd, 1H, ^3^*J*=9.1 Hz, ^2^*J*=14 Hz, CH_2_Ph), 3.37 (dd, 1H, ^3^*J*=9.1 Hz, ^2^*J*=14 Hz, CH_2_Ph), 3.66 (s, 3H, OMe), 4.53 (m, 1H, CH_chiral_), 5.60 (s, 1H, =CH), 7.22–7.33 (m, 5H, Ph), 10.9 (d, 1H, ^3^*J* = 10 Hz, NH) p.p.m.; ^13^C NMR (100 MHz, DMSO-D_6_): δ 26.8, 31.5, 39.9, 52.2, 58.1, 86.0, 96.9, 127.5, 128.9, 129.5, 135.4, 169.9, 172.0, 173.2, 181.2 p.p.m.. Crystals were grown from a methanol solution, which was slowly evaporated at room temperature.

### 3. Refinement

All H atoms attached to carbon were positioned with idealized geometry and were refined isotropically. For H atoms of CH_3_ group, *U*_iso_(H) was set to 1.5*U*_eq_(C) using a riding model with C—H = 0.96 Å. For all remaining H atoms attached to C atoms, *U*_iso_(H) was set to 1.2*U*_eq_(C) using a riding model with the following C—H distances: C—H (CH) = 0.93 Å, C—H (CH_chiral_) = 0.98 Å and C—H (CH_2_) = 0.97 Å. H atoms attached to nitrogen, H atoms of the water molecule and the H atom of the carboxylic acid fragment were located in difference Fourier maps, and were refined with *U*_iso_ values set to 1.5*U*_eq_ of the parent atom. Reflections (001) and (001) were omitted due to the large difference observed between *F_o_*^2^ and Fc^2^.

### Crystal data

**Table:**

C_17_H_18_Cl_3_NO_5_·H_2_O	*F*(000) = 228
*M**_r_* = 440.69	*D*_x_ = 1.461 Mg m^−^^3^
Triclinic, *P*1	Melting point: 393 K
*a* = 5.6684 (16) Å	Mo *K*α radiation, λ = 0.71073 Å
*b* = 8.601 (3) Å	Cell parameters from 3866 reflections
*c* = 10.336 (3) Å	θ = 3.0–25.5°
α = 87.720 (19)°	µ = 0.49 mm^−^^1^
β = 85.696 (17)°	*T* = 296 K
γ = 85.649 (17)°	Blade, colorless
*V* = 500.8 (2) Å^3^	0.98 × 0.30 × 0.12 mm
*Z* = 1	

### Data collection

**Table:**

Bruker APEXII CCD diffractometer	6020 independent reflections
Radiation source: fine-focus sealed tube	4784 reflections with *I* > 2σ(*I*)
Graphite monochromator	*R*_int_ = 0.024
φ and ω scans	θ_max_ = 30.7°, θ_min_ = 2.4°
Absorption correction: gaussian (*XPREP*; Bruker, 2006)	*h* = −8→8
*T*_min_ = 0.881, *T*_max_ = 1	*k* = −12→12
13424 measured reflections	*l* = −14→14

### Refinement

**Table:**

Refinement on *F*^2^	Hydrogen site location: mixed
Least-squares matrix: full	H atoms treated by a mixture of independent and constrained refinement
*R*[*F*^2^ > 2σ(*F*^2^)] = 0.040	*w* = 1/[σ^2^(*F*_o_^2^) + (0.0502*P*)^2^ + 0.0376*P*] where *P* = (*F*_o_^2^ + 2*F*_c_^2^)/3
*wR*(*F*^2^) = 0.105	(Δ/σ)_max_ < 0.001
*S* = 1.04	Δρ_max_ = 0.41 e Å^−^^3^
6020 reflections	Δρ_min_ = −0.32 e Å^−^^3^
256 parameters	Absolute structure: Flack parameter determined using 1984 quotients [(*I*^+^)-(*I*^-^)]/[(*I*^+^)+(*I*^-^)] (Parsons *et al.*, 2013)
3 restraints	Absolute structure parameter: 0.04 (2)

### Special details

**Table:**

Experimental. Absorption correction: XPREP (Bruker, 2006) was used to perform the Gaussian absorption correction based on the face-indexed crystal size.
Geometry. All e.s.d.'s (except the e.s.d. in the dihedral angle between two l.s. planes) are estimated using the full covariance matrix. The cell e.s.d.'s are taken into account individually in the estimation of e.s.d.'s in distances, angles and torsion angles; correlations between e.s.d.'s in cell parameters are only used when they are defined by crystal symmetry. An approximate (isotropic) treatment of cell e.s.d.'s is used for estimating e.s.d.'s involving l.s. planes.

### Fractional atomic coordinates and isotropic or equivalent isotropic displacement parameters (Å^2^)

**Table:**

	*x*	*y*	*z*	*U*_iso_*/*U*_eq_	
O33	0.4813 (5)	1.0147 (3)	0.9230 (3)	0.0554 (6)	
H33B	0.629 (11)	0.977 (6)	0.919 (5)	0.083*	
H41	0.147 (9)	0.465 (6)	0.794 (5)	0.083*	
H33A	0.458 (10)	1.092 (7)	0.887 (5)	0.083*	
H92	0.352 (9)	0.869 (6)	0.864 (5)	0.083*	
Cl1	0.75049 (16)	0.24572 (10)	0.42096 (9)	0.0652 (3)	
Cl2	0.50084 (18)	0.01516 (11)	0.57338 (11)	0.0759 (3)	
Cl3	0.88358 (15)	0.16349 (14)	0.67617 (11)	0.0742 (3)	
N41	0.1053 (4)	0.5403 (3)	0.7462 (2)	0.0403 (5)	
C1	0.6395 (5)	0.1924 (3)	0.5785 (3)	0.0432 (6)	
C7	−0.1436 (5)	0.7761 (4)	0.3642 (3)	0.0424 (6)	
C9	0.0625 (5)	0.7901 (3)	0.8625 (3)	0.0439 (6)	
C3	0.3699 (5)	0.4434 (3)	0.5739 (3)	0.0412 (6)	
H3	0.4282	0.4569	0.4880	0.049*	
C10	−0.0576 (5)	0.6530 (3)	0.8145 (3)	0.0402 (6)	
H10	−0.1704	0.6956	0.7526	0.048*	
C5	0.1234 (5)	0.6912 (3)	0.5398 (3)	0.0414 (6)	
H5A	0.2526	0.7135	0.4764	0.050*	
H5B	0.0854	0.7828	0.5913	0.050*	
C111	−0.0514 (5)	0.4758 (4)	1.0193 (3)	0.0455 (6)	
C2	0.4540 (5)	0.3128 (3)	0.6460 (3)	0.0386 (5)	
C6	−0.0917 (6)	0.6567 (4)	0.4700 (3)	0.0472 (7)	
H6A	−0.0645	0.5545	0.4328	0.057*	
H6B	−0.2285	0.6543	0.5321	0.057*	
C11	−0.2023 (5)	0.5712 (4)	0.9264 (3)	0.0475 (7)	
H11A	−0.3005	0.6496	0.9745	0.057*	
H11B	−0.3071	0.5033	0.8897	0.057*	
C112	0.0981 (7)	0.5453 (4)	1.0959 (3)	0.0584 (8)	
H112	0.1040	0.6532	1.0916	0.070*	
C8	−0.4279 (7)	0.8699 (5)	0.2188 (4)	0.0649 (10)	
H8A	−0.5833	0.8501	0.1953	0.097*	
H8B	−0.3165	0.8573	0.1446	0.097*	
H8C	−0.4299	0.9745	0.2481	0.097*	
C116	−0.0616 (8)	0.3156 (4)	1.0306 (4)	0.0638 (9)	
H116	−0.1627	0.2664	0.9813	0.077*	
C115	0.0793 (10)	0.2279 (5)	1.1156 (4)	0.0792 (13)	
H115	0.0718	0.1202	1.1222	0.095*	
C113	0.2391 (9)	0.4563 (6)	1.1789 (4)	0.0734 (11)	
H113	0.3422	0.5039	1.2280	0.088*	
C114	0.2259 (9)	0.2964 (6)	1.1884 (4)	0.0774 (13)	
H114	0.3183	0.2363	1.2450	0.093*	
O72	−0.3587 (4)	0.7608 (3)	0.3218 (2)	0.0535 (5)	
O92	0.2915 (4)	0.7852 (3)	0.8366 (3)	0.0554 (5)	
O91	−0.0517 (4)	0.8944 (3)	0.9190 (3)	0.0591 (6)	
O71	−0.0124 (5)	0.8698 (3)	0.3220 (3)	0.0637 (7)	
C4	0.2004 (5)	0.5547 (3)	0.6269 (3)	0.0370 (5)	
O21	0.3934 (4)	0.2806 (2)	0.7613 (2)	0.0473 (5)	

### Atomic displacement parameters (Å^2^)

**Table:**

	*U*^11^	*U*^22^	*U*^33^	*U*^12^	*U*^13^	*U*^23^
O33	0.0494 (13)	0.0492 (13)	0.0673 (15)	−0.0017 (11)	−0.0076 (11)	0.0051 (11)
Cl1	0.0759 (6)	0.0560 (5)	0.0586 (5)	0.0031 (4)	0.0241 (4)	−0.0061 (4)
Cl2	0.0741 (6)	0.0476 (4)	0.1050 (8)	−0.0204 (4)	0.0308 (5)	−0.0288 (5)
Cl3	0.0413 (4)	0.0946 (7)	0.0838 (6)	0.0154 (4)	−0.0058 (4)	−0.0032 (5)
N41	0.0439 (13)	0.0350 (11)	0.0398 (12)	0.0079 (10)	−0.0005 (9)	0.0012 (9)
C1	0.0375 (14)	0.0379 (14)	0.0531 (16)	−0.0024 (11)	0.0056 (11)	−0.0056 (11)
C7	0.0410 (14)	0.0435 (15)	0.0412 (14)	0.0029 (12)	−0.0024 (11)	0.0028 (11)
C9	0.0441 (14)	0.0397 (14)	0.0459 (15)	0.0077 (11)	−0.0048 (11)	0.0056 (12)
C3	0.0417 (14)	0.0403 (14)	0.0398 (13)	0.0013 (11)	0.0024 (10)	0.0017 (11)
C10	0.0375 (13)	0.0376 (13)	0.0444 (14)	0.0073 (11)	−0.0052 (11)	−0.0035 (11)
C5	0.0419 (14)	0.0338 (13)	0.0481 (15)	−0.0020 (10)	−0.0039 (11)	0.0043 (11)
C111	0.0480 (15)	0.0477 (16)	0.0385 (13)	0.0000 (13)	0.0078 (12)	−0.0018 (12)
C2	0.0349 (12)	0.0363 (13)	0.0440 (14)	0.0022 (10)	−0.0012 (10)	−0.0049 (10)
C6	0.0478 (16)	0.0414 (15)	0.0532 (16)	−0.0051 (12)	−0.0114 (13)	0.0081 (13)
C11	0.0387 (14)	0.0508 (17)	0.0516 (16)	0.0032 (12)	0.0001 (12)	−0.0028 (13)
C112	0.075 (2)	0.0510 (19)	0.0489 (17)	−0.0038 (17)	−0.0081 (16)	0.0032 (14)
C8	0.063 (2)	0.081 (3)	0.0495 (18)	0.0133 (19)	−0.0149 (15)	0.0064 (17)
C116	0.082 (3)	0.0495 (19)	0.059 (2)	−0.0072 (17)	0.0021 (18)	−0.0027 (15)
C115	0.115 (4)	0.049 (2)	0.069 (3)	0.007 (2)	0.006 (3)	0.0063 (18)
C113	0.084 (3)	0.085 (3)	0.052 (2)	−0.004 (2)	−0.0171 (19)	0.0069 (19)
C114	0.093 (3)	0.078 (3)	0.055 (2)	0.026 (2)	−0.002 (2)	0.013 (2)
O72	0.0482 (12)	0.0614 (14)	0.0511 (12)	−0.0012 (10)	−0.0121 (9)	0.0063 (10)
O92	0.0449 (12)	0.0501 (13)	0.0709 (14)	−0.0013 (10)	−0.0017 (10)	−0.0073 (11)
O91	0.0540 (13)	0.0445 (12)	0.0778 (16)	0.0107 (10)	−0.0050 (11)	−0.0166 (11)
O71	0.0538 (14)	0.0628 (15)	0.0738 (16)	−0.0076 (11)	−0.0098 (12)	0.0244 (12)
C4	0.0379 (12)	0.0331 (13)	0.0400 (13)	−0.0025 (10)	−0.0037 (10)	0.0014 (10)
O21	0.0524 (12)	0.0423 (11)	0.0432 (11)	0.0133 (9)	0.0038 (8)	0.0029 (9)

### Geometric parameters (Å, º)

**Table:**

O33—H33B	0.87 (6)	C5—H5B	0.9700
O33—H33A	0.76 (6)	C111—C116	1.384 (5)
Cl1—C1	1.757 (3)	C111—C112	1.385 (5)
Cl2—C1	1.772 (3)	C111—C11	1.510 (4)
Cl3—C1	1.770 (3)	C2—O21	1.241 (4)
N41—C4	1.314 (4)	C6—H6A	0.9700
N41—C10	1.453 (3)	C6—H6B	0.9700
N41—H41	0.83 (5)	C11—H11A	0.9700
C1—C2	1.564 (4)	C11—H11B	0.9700
C7—O71	1.186 (4)	C112—C113	1.385 (5)
C7—O72	1.343 (4)	C112—H112	0.9300
C7—C6	1.500 (4)	C8—O72	1.448 (4)
C9—O91	1.209 (4)	C8—H8A	0.9600
C9—O92	1.303 (4)	C8—H8B	0.9600
C9—C10	1.523 (4)	C8—H8C	0.9600
C3—C2	1.397 (4)	C116—C115	1.393 (6)
C3—C4	1.401 (4)	C116—H116	0.9300
C3—H3	0.9300	C115—C114	1.344 (7)
C10—C11	1.545 (4)	C115—H115	0.9300
C10—H10	0.9800	C113—C114	1.382 (7)
C5—C4	1.509 (4)	C113—H113	0.9300
C5—C6	1.517 (4)	C114—H114	0.9300
C5—H5A	0.9700	O92—H92	0.89 (5)
			
H33B—O33—H33A	115 (6)	C7—C6—H6A	109.2
C4—N41—C10	126.9 (2)	C5—C6—H6A	109.2
C4—N41—H41	121 (4)	C7—C6—H6B	109.2
C10—N41—H41	112 (4)	C5—C6—H6B	109.2
C2—C1—Cl1	116.0 (2)	H6A—C6—H6B	107.9
C2—C1—Cl3	107.9 (2)	C111—C11—C10	113.8 (2)
Cl1—C1—Cl3	107.55 (16)	C111—C11—H11A	108.8
C2—C1—Cl2	107.0 (2)	C10—C11—H11A	108.8
Cl1—C1—Cl2	109.06 (17)	C111—C11—H11B	108.8
Cl3—C1—Cl2	109.22 (17)	C10—C11—H11B	108.8
O71—C7—O72	124.4 (3)	H11A—C11—H11B	107.7
O71—C7—C6	125.1 (3)	C111—C112—C113	120.9 (4)
O72—C7—C6	110.5 (2)	C111—C112—H112	119.6
O91—C9—O92	124.1 (3)	C113—C112—H112	119.6
O91—C9—C10	121.0 (3)	O72—C8—H8A	109.5
O92—C9—C10	114.9 (3)	O72—C8—H8B	109.5
C2—C3—C4	122.1 (3)	H8A—C8—H8B	109.5
C2—C3—H3	119.0	O72—C8—H8C	109.5
C4—C3—H3	119.0	H8A—C8—H8C	109.5
N41—C10—C9	113.6 (2)	H8B—C8—H8C	109.5
N41—C10—C11	110.4 (2)	C111—C116—C115	120.1 (4)
C9—C10—C11	111.3 (2)	C111—C116—H116	119.9
N41—C10—H10	107.1	C115—C116—H116	119.9
C9—C10—H10	107.1	C114—C115—C116	121.0 (4)
C11—C10—H10	107.1	C114—C115—H115	119.5
C4—C5—C6	110.9 (2)	C116—C115—H115	119.5
C4—C5—H5A	109.5	C114—C113—C112	119.7 (4)
C6—C5—H5A	109.5	C114—C113—H113	120.1
C4—C5—H5B	109.5	C112—C113—H113	120.1
C6—C5—H5B	109.5	C115—C114—C113	119.9 (4)
H5A—C5—H5B	108.0	C115—C114—H114	120.1
C116—C111—C112	118.3 (3)	C113—C114—H114	120.1
C116—C111—C11	120.3 (3)	C7—O72—C8	115.5 (3)
C112—C111—C11	121.4 (3)	C9—O92—H92	111 (3)
O21—C2—C3	125.9 (3)	N41—C4—C3	122.1 (2)
O21—C2—C1	115.3 (2)	N41—C4—C5	120.6 (2)
C3—C2—C1	118.7 (3)	C3—C4—C5	117.4 (2)
C7—C6—C5	112.0 (2)		
			
C4—N41—C10—C9	−76.6 (4)	N41—C10—C11—C111	54.1 (3)
C4—N41—C10—C11	157.5 (3)	C9—C10—C11—C111	−73.0 (3)
O91—C9—C10—N41	178.7 (3)	C116—C111—C112—C113	1.9 (6)
O92—C9—C10—N41	−0.6 (4)	C11—C111—C112—C113	−178.7 (4)
O91—C9—C10—C11	−56.0 (3)	C112—C111—C116—C115	−1.1 (5)
O92—C9—C10—C11	124.7 (3)	C11—C111—C116—C115	179.5 (4)
C4—C3—C2—O21	−0.5 (5)	C111—C116—C115—C114	0.3 (7)
C4—C3—C2—C1	178.8 (3)	C111—C112—C113—C114	−1.9 (7)
Cl1—C1—C2—O21	−173.6 (2)	C116—C115—C114—C113	−0.3 (7)
Cl3—C1—C2—O21	−53.0 (3)	C112—C113—C114—C115	1.0 (7)
Cl2—C1—C2—O21	64.5 (3)	O71—C7—O72—C8	−0.8 (5)
Cl1—C1—C2—C3	7.0 (4)	C6—C7—O72—C8	−179.7 (3)
Cl3—C1—C2—C3	127.7 (3)	C10—N41—C4—C3	175.1 (3)
Cl2—C1—C2—C3	−114.9 (3)	C10—N41—C4—C5	−7.0 (4)
O71—C7—C6—C5	13.3 (5)	C2—C3—C4—N41	−2.2 (5)
O72—C7—C6—C5	−167.8 (3)	C2—C3—C4—C5	179.8 (3)
C4—C5—C6—C7	−168.9 (3)	C6—C5—C4—N41	−86.6 (3)
C116—C111—C11—C10	−115.5 (3)	C6—C5—C4—C3	91.4 (3)
C112—C111—C11—C10	65.1 (4)		

### Hydrogen-bond geometry (Å, º)

**Table:**

*D*—H···*A*	*D*—H	H···*A*	*D*···*A*	*D*—H···*A*
C6—H6*A*···Cl1^i^	0.97	2.94	3.774 (3)	145
O33—H33*B*···O91^ii^	0.87 (6)	1.89 (6)	2.766 (4)	177 (5)
N41—H41···O21	0.83 (5)	2.05 (6)	2.672 (3)	131 (5)
O33—H33*A*···O21^iii^	0.76 (6)	2.06 (6)	2.815 (3)	171 (6)
O92—H92···O33	0.89 (5)	1.66 (5)	2.542 (4)	175 (5)
C3—H3···Cl1	0.93	2.55	3.031 (3)	112

Symmetry codes: (i) *x*−1, *y*, *z*; (ii) *x*+1, *y*, *z*; (iii) *x*, *y*+1, *z*.
